# The Regulatory Roles of PPARs in Skeletal Muscle Fuel Metabolism and Inflammation: Impact of PPAR Agonism on Muscle in Chronic Disease, Contraction and Sepsis

**DOI:** 10.3390/ijms22189775

**Published:** 2021-09-10

**Authors:** Hannah Crossland, Dumitru Constantin-Teodosiu, Paul L. Greenhaff

**Affiliations:** 1 MRC/Versus Arthritis Centre for Musculoskeletal Ageing Research, Division of Physiology, Pharmacology and Neuroscience, School of Life Sciences, Queen’s Medical Centre, University of Nottingham, Nottingham NG7 2UH, UK; mbzhc@exmail.nottingham.ac.uk (H.C.); tim.constantin@nottingham.ac.uk (D.C.-T.); 2 National Institute for Health Research Nottingham Biomedical Research Centre, Queen’s Medical Centre, University of Nottingham, Nottingham NG7 2UH, UK

**Keywords:** skeletal muscle, inflammation, PPARs, substrate metabolism

## Abstract

The peroxisome proliferator-activated receptor (PPAR) family of transcription factors has been demonstrated to play critical roles in regulating fuel selection, energy expenditure and inflammation in skeletal muscle and other tissues. Activation of PPARs, through endogenous fatty acids and fatty acid metabolites or synthetic compounds, has been demonstrated to have lipid-lowering and anti-diabetic actions. This review will aim to provide a comprehensive overview of the functions of PPARs in energy homeostasis, with a focus on the impacts of PPAR agonism on muscle metabolism and function. The dysregulation of energy homeostasis in skeletal muscle is a frequent underlying characteristic of inflammation-related conditions such as sepsis. However, the potential benefits of PPAR agonism on skeletal muscle protein and fuel metabolism under these conditions remains under-investigated and is an area of research opportunity. Thus, the effects of PPARγ agonism on muscle inflammation and protein and carbohydrate metabolism will be highlighted, particularly with its potential relevance in sepsis-related metabolic dysfunction. The impact of PPARδ agonism on muscle mitochondrial function, substrate metabolism and contractile function will also be described.

## 1. Introduction

Peroxisome proliferator-activated receptors (PPARs) are a group of transcription factors implicated in wide-ranging cellular functions, including lipid metabolism, inflammatory responses and cell proliferation and differentiation [1]. Three PPAR subtypes exist (PPARα, PPARδ and PPARγ). They are activated in vivo by endogenous fatty acids and their metabolites and synthetic compounds developed for their lipid-lowering and anti-diabetic actions. Skeletal muscle is a tissue that displays high metabolic flexibility, comprising different fibre types that vary according to their contractile and metabolic properties [2]. For example, slow-twitch type I fibres have a relatively high capillary density, are rich in mitochondria and possess a relatively high capacity for oxidative metabolism during contraction. In contrast, fast-twitch type IIx fibres have a lower capillary density and a high capacity for energy delivery from non-mitochondrial routes during contraction. Disturbances in skeletal muscle energy homeostasis play a key part in the pathogenesis of several chronic non-communicable disease conditions, including type 2 diabetes (T2D) and chronic lung disease. The dysregulation of skeletal muscle energy homeostasis is also a frequent underlying characteristic of acute inflammation-related conditions, such as sepsis [3] and surgical trauma. The role of PPAR agonism in modulating skeletal muscle protein and fuel metabolism in these conditions is relatively poorly understood. Still, the potential of such an approach will be addressed in this article. Specifically, this review will aim to provide an overview of the metabolic regulatory roles of PPARs in energy homeostasis, with a focus on the impacts of PPARδ agonism on skeletal muscle metabolism and contractile function, primarily highlighting studies that have involved in vivo/ex vivo animal models or human volunteers. Furthermore, the focus will be directed towards the potential role of PPARγ agonism in alleviating muscle inflammation and metabolic disturbances during sepsis. 

## 2. Metabolic Functions of PPARs and Their Actions in Skeletal Muscle

Peroxisome proliferator-activated receptors (PPARs) are a group of proteins that belong to the nuclear hormone receptor superfamily of ligand-activated transcription factors. PPAR transcriptional activity is mediated by heterodimers of PPARs with retinoid X receptor (RXR), which subsequently bind to DNA sequence elements (PPREs) in regulatory regions of target genes [4]. Three PPAR subtypes have been identified (PPARα, PPARδ (also known as PPARβ) and PPARγ [5]. Through their interactions with endogenous lipids and lipid metabolites, PPARs have been reported to regulate many metabolic processes, including lipid and glucose homeostasis, cell proliferation and inflammation [1]. Several endogenous compounds, including n-3 and n-6 fatty acids, eicosanoids and phospholipids, have been identified as natural ligands of PPARs. In addition to this, activation of PPAR activity by pharmacological agonists has been identified as a promising treatment strategy for conditions related to insulin resistance and dyslipidaemia, in part through increased fatty acid oxidation in skeletal muscle, thereby decreasing overall body fat content [6]. 

Each PPAR subtype has been attributed to different tissue-specific expression levels and functions. For example, PPARα is highly expressed in tissue types that undergo significant fatty acid catabolism, such as brown adipose tissue, heart and liver [7]. Activated by polyunsaturated fatty acids (PUFA) and leukotriene, PPARα has an important function in fatty acid catabolism and carbohydrate metabolism [8,9]. Synthetic compounds that act as agonists of PPARα are known as fibrates, whose actions are important in lipid-lowering activities and cardio-protection [10,11]. PPARγ, on the other hand, is variably expressed in adipocytes, macrophages, placenta and other tissues and is activated by specific endogenous fatty acid metabolites (such as 15-deoxy-prostaglandin J2) as well as by a class of insulin sensitisers known as thiazolidinediones (TZDs) [12]. TZDs have been proven to be important in the treatment of T2D. While early TZDs (e.g., Troglitazone) were related to severe hepatic side effects, other newer available TZDs (Rosiglitazone, Pioglitazone) are not toxic to the liver. PPARγ plays a central role in adipogenesis, whereby the insulin-sensitising effect of TZDs may be due to new adipose cell recruitment, enabling increased lipid storage capacity and adipokine secretion [13]. PPARγ activation also regulates the transcription of genes that promote the synthesis of triglycerides [13]. In patients with T2D, administration of TZDs successfully improved insulin-stimulated glucose disposal under euglycaemic-hyperinsulinaemic clamp conditions [14,15], where skeletal muscle plays a central glucose-lowering role. One mechanism by which TZDs exert their insulin sensitising actions on skeletal muscle is through the modulation of adipose secretory factors, such as adiponectin. Increased secretion of adiponectin has been suggested to act as an insulin sensitiser for liver and skeletal muscle, and this occurs through the activation of PPARγ [16]. 

The role of PPARδ has remained relatively unclear until recently, where it has been associated with a wide range of metabolic functions in vivo [17,18]. It has broad expression across tissues and is activated by various ligands, such as long-chain fatty acids. A developmental regulatory role has been identified for PPARδ, as well as regulation of lipid metabolism [17,18]. It is the predominant isotype in skeletal muscle, where it has been linked to fuel metabolism, energy expenditure, inflammation, and fibre type switching through physical exercise [17,19]. Both PPARα and PPARδ have been demonstrated to regulate genes for proteins involved in fatty acid uptake and oxidation, including lipoprotein lipase (LPL), fatty acid-binding protein 3 (FABP), stearoyl-Coenzyme A desaturase (SCD)-1 and cluster of differentiation 36 (CD36) [20,21]. During fasting, PPARδ expression is upregulated in rodent skeletal muscles, which is important in regulating the cellular uptake and oxidation of free fatty acids (FFA) as an energy source for ATP production [22]. 

Through their importance in metabolic regulation, the role of all three PPAR subtypes in skeletal muscle metabolism has been established. For example, one link between PPARs and metabolic regulation in skeletal muscle appears to be through the upregulation of pyruvate dehydrogenase kinase 4 (PDK4), a key regulator of the pyruvate dehydrogenase complex (PDC). The PDC activation status is regulated by various competing PDKs and pyruvate dehydrogenase phosphatase (PDP) proteins [23]. These covalent processes ultimately determine the extent of PDC phosphorylation (i.e., activation). There are four isoforms of PDK (PDK1-4) and two isoforms of PDP (PDP1 and 2) [24,25]. While PDK1 and PDK3 appear to be mainly expressed in the heart, pancreatic islet cells and kidney, PDK2 and PDK4 are expressed in most tissues, including heart and skeletal muscle [24]. Selective PDK4 upregulation has been demonstrated in response to starvation conditions and pathologies such as T2D [26,27], which is thought to be due to changes in FFA availability in skeletal muscle. An increase in fatty acid oxidation via PPARδ agonism [6], and starvation [28], is believed to be responsible for the PDK4 transcriptional activation, thereby inactivating PDC (the rate-limiting enzyme in mitochondrial carbohydrate oxidation). It should be noted, however, that a lack of association between increases in plasma FFA levels and muscle PDK4 expression has been reported during fasting in humans [29], with no observable changes in muscle PPARα expression, indicating that other factors could also be important in PDK4 upregulation. One such factor could be the Forkhead box class O (FOXO) family of transcription factors, which has been linked to promoter binding of the PDK4 gene as a result of FFA-mediated nuclear translocation [30]. 

In addition to increased availability of endogenous fatty acids and their metabolites being associated with PPAR activation, inflammation has been proven to be a major site of PPAR regulation, which can occur through both direct and indirect mechanisms [31]. As mentioned, PPARs have emerged as targets of drugs used to treat various aspects of the metabolic syndrome, of which inflammation is an underlying key factor. All three PPAR isotypes have been shown to exert anti-inflammatory effects during conditions of chronic low-grade inflammation, characterised by increased circulatory cytokines and acute-phase proteins [32,33]. PPARα was shown to upregulate the expression of IkB, a factor that suppresses the nuclear translocation and transcriptional activity of the pro-inflammatory nuclear factor kappa-light-chain-enhancer of activated B cells (NF-kB) [34]. PPARγ has also been shown to reduce activation of NF-kB, as well as inhibit pro-inflammatory cytokine production in T lymphocytes and induction of anti-inflammatory regulatory molecules of the innate immune system [35]. 

In summary, all three PPAR subtypes have distinct yet overlapping roles in regulating metabolic function and inflammation (see Table 1), and synthetic compounds aimed at activating the PPARs have been developed for their lipid-lowering and anti-diabetic actions. In skeletal muscle, PPAR activation appears important in the upregulation of PDK4, thereby demonstrating its essential role in regulating carbohydrate oxidation and energy homeostasis. The following section of this review will focus in more detail on the impact of PPARδ agonism on muscle metabolism and contractile function and PPARγ agonism on muscle metabolism and inflammation.

## 3. PPARδ Agonism and Skeletal Muscle Metabolism, Contractile Function and Inflammation

**Figure 1:**
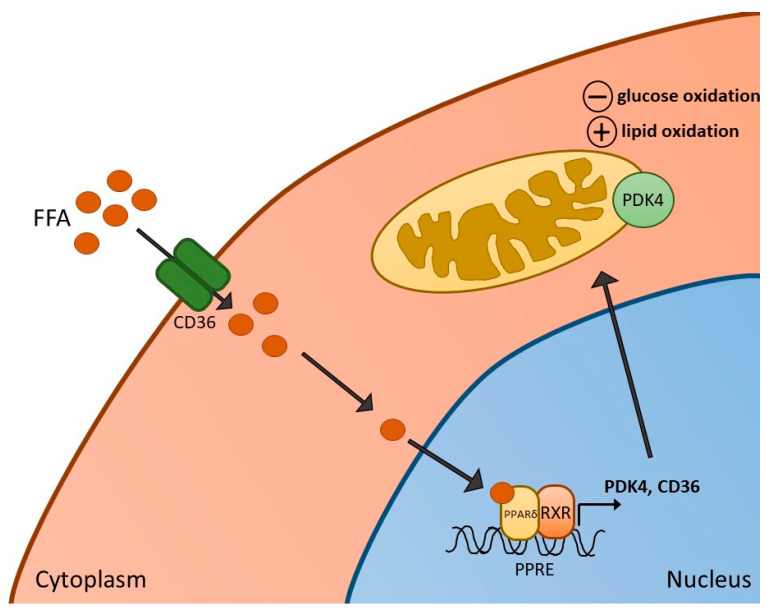
Activation of PPARδ in skeletal muscle. Increased free fatty acids (FFA) and their metabolites enter skeletal muscle via the FFA transporter CD36, resulting in the formation of a heterodimer of PPARδ and retinoid X receptor (RXR), and subsequent activation of PPARδ-dependent genes, such as pyruvate dehydrogenase kinase 4 (PDK4) (and CD36 itself). Activation of PDK4 can result in reduced rates of glucose oxidation as well as increased fatty acid oxidation in mitochondria.

Several in vivo animal studies have been performed with the aim of determining the impact of PPARδ agonism on skeletal muscle metabolism and function. We previously demonstrated in our laboratory that 6 days of administration of the PPARδ agonist, GW610742 [36], resulted in increased activity of β-hydroxy acyl-CoA dehydrogenase (β-HAD) in resting rat soleus muscle, which is a key step in β-oxidation in the mitochondria. Compared with control animals, these changes were paralleled by increased expression of muscle PDK2 and PDK4 mRNA and PDK4 protein expression. Thus, evidence points towards PPAR activation in skeletal muscle being, in part, important in mediating FFA-induced PDK4 upregulation in skeletal muscle, thereby contributing to PDC inhibition, suppressing PDC-regulated carbohydrate oxidation, and switching fuel selection towards fat oxidation in skeletal muscle (Figure 1). We also measured the impact of GW610742 on muscle growth-related pathways since FOXO1, which plays a part in PDK4 upregulation, has also been suggested to increase transcription of MAFbx and MuRF1, thereby activating ubiquitin-proteasome mediated muscle proteolysis [37]. In keeping with this, administration of the PPARδ agonist resulted in increases in muscle mRNA and protein expression of MAFbx and MuRF1, suggesting that potentially the induction of muscle atrophy signalling is another consequence of PPARδ agonism. Collectively, the findings pointed to PPARδ agonism being involved in the regulation of muscle fuel selection and the induction of a muscle atrophy programme via a single common signalling pathway. It should be stated, however, there was no evidence of soleus muscle atrophy based on the muscle protein:DNA ratio after 6 days of GW610742 administration compared with control.

In line with the above findings relating to a PPARδ agonism induced switch in muscle fuel selection away from carbohydrate to increased fat oxidation, in another study, mice treated with the PPARδ agonist GW501516 exhibited increased PGC-1α levels, and improved prolonged low-intensity wheel-running performance. They also saw hypertrophy of oxidative slow-twitch myofibres, which are rich in mitochondria, perhaps suggesting increased reliance on the catabolism of FA through mitochondrial beta-oxidation [38]. However, we further reported that when muscle contraction was increased to an intensity that necessitates carbohydrate to become an obligate fuel for contraction, PPARδ agonism negatively affected contractile function in rats [39]. Specifically, male Wistar rats received the PPARδ agonist GW610742X (or vehicle) for 6 days. The gastrocnemius–soleus–plantaris muscle group was isolated and subjected to submaximal electrically evoked contraction using a perfused hindlimb model. The contraction intensity was fixed to guarantee carbohydrate become an essential fuel, and PDC activity was increased, ensuring pyruvate derived acetyl group delivery to the mitochondrion [40]. We observed that PDC activity during contraction was significantly less with the PPARδ agonist than control, while anaerobic metabolism (reflected by phosphocreatine hydrolysis and lactate accumulation) was greater. We proposed that this collectively accounted for the observed impaired contractile function with GW610742X agonist, indicating that PPARδ agonism can impair the contractile muscle function by inhibiting carbohydrate oxidation during muscle contraction where carbohydrate is an obligate fuel. 

We have also sought to determine whether PPAR transcription factors may be necessary for the high-fat feeding induced inhibition of PDC activation and carbohydrate oxidation during submaximal exercise in humans [41,42]. Healthy male volunteers were given a control diet or an iso-caloric high-fat diet (HFD). They underwent 60 min of submaximal exercise at an intensity equivalent to 75% maximal oxygen uptake. There was a relative increase in expression of PDK4 in muscle with HFD compared to control, alongside reduced PDC activation in muscle. Exercise increased PDC activity and carbohydrate utilisation with both diets, but these measures were diminished with the HFD. In terms of PPAR expression, there was no effect of the high-fat diet on the mRNA expression of PPARδ. However, PPARγ and PPARα were increased at rest, though this increase was not apparent during exercise. Of note, the expression of PPARα mRNA was lower in another group of volunteers that underwent a HFD but were also treated with dichloroacetate (DCA), a potent inhibitor of PDK2 and PDK4 and, therefore, a stimulator of PDC activity, which restored carbohydrate oxidation during exercise in this group. Thus, in humans, these results appear to suggest there may not be significant involvement of PPARs in increasing muscle PDK4 expression, although muscle protein levels of each PPAR were not measured. 

Following these findings, further work from our laboratory studied the role of PPARδ (and FOXO1) in palmitate-induced PDC inhibition and carbohydrate use using a skeletal muscle cell model [43]. Myotubes were treated with palmitate for 16 hrs in the presence or absence of continuous electrical pulse stimulation, the latter having been shown to increase glucose uptake and carbohydrate oxidation in muscle cells [44,45], and therefore potentially having the capacity to reverse palmitate-mediated inhibition of PDC. It was observed that palmitate reduced glucose uptake, PDC activity and maximal rates of palmitate derived mitochondrial ATP production whilst also increasing PDK4, PPARδ and PPARα proteins. There was also a significant reduction in the magnitude of FOXO1 phosphorylation, indicating its nuclear translocation and subsequent activation. Electrical pulse stimulation reversed many palmitate-induced changes to carbohydrate oxidation and was associated with reduced PDK4 protein and reduced PPARδ (but not PPARα) protein content. Collectively, while more work is required to elucidate the relative importance of PPARδ and FOXO1 transcription factors in mediating PDK4 transcription, particularly in humans, these findings indicate their potential roles in palmitate-induced impairments in PDC activity and carbohydrate oxidation in skeletal muscle. 

As discussed earlier, all three PPAR subtypes have the potential for treating inflammatory states, with their anti-inflammatory effects well characterised [31,32]. Based upon this, targeting PPARs could potentially represent an attractive avenue for alleviating inflammation and metabolic disturbances during conditions such as sepsis. Fibrates are proven to be beneficial in treating dyslipidaemia. They may lower circulating triglyceride levels in the blood by inducing hepatic fatty acid oxidation and increasing levels of high-density lipoproteins [10,11]. TZDs have also proven antidiabetic effects, though their clinical use and development has been limited due to adverse side effects (increased risk of congestive heart failure, weight gain, increased risk of bone fracture). However, inflammation associated with the pathophysiology of T2D is typically chronic and low-grade, while sepsis is an acute, high-grade inflammatory condition, which may increase their utility in this scenario. Sepsis is characterised by an uncontrolled host response to an infection and remains a major cause of morbidity (including muscle atrophy and insulin resistance) and mortality worldwide [46]. Ongoing work aims to understand its pathophysiology and develop novel therapeutics since current available therapeutic strategies remain limited. In patients with sepsis, severe metabolic dysregulation occurs, which contributes to sepsis pathophysiology and resultant organ failure. It has been observed that early and rapid skeletal muscle wasting can occur with critical illness, which can play a major role in causing the increased length of hospital stay and delayed recovery [3]. In conjunction with this, trials aimed at attenuating muscle wasting and improving physical function through either nutritional support or exercise approaches have proved inconsistent. 

It is unclear what causes the decline in muscle protein synthesis early in critical illness and could feasibly be related to impaired mitochondrial function or tissue content [47]. Decreased substrate utilisation, including both carbohydrate and fatty acids, is a consequence of critical illness and could result in impaired metabolic function in muscle [48]. Infusion or injection of the bacterial endotoxin lipopolysaccharide (LPS) can reproduce many metabolic changes seen in sepsis [49]. The endotoxemia model, therefore, represents a relevant physiological model of sepsis. In a rat model of LPS-induced septic shock, tissue protein expression (renal and cardiac) of cytosolic and nuclear PPARα, PPARδ and PPARγ and nuclear translocation of these proteins were decreased with LPS [50]. In a rodent model of septic shock, early administration of a selective RXR agonist (bexarotene) was shown to prevent LPS-induced decrease in mean arterial pressure, as well as LPS-induced decreases in tissue PPARα/δ/γ-RXRα heterodimer formation [51]. The concurrent decline in circulating iNOS and LDH levels led the authors to conclude that activation of PPARα/δ/γ-RXRα heterodimers contributes to the beneficial effect of bexarotene to prevent the hypotension associated with inflammation and tissue injury during rat endotoxemia. 

In terms of muscle-specific effects of PPARδ agonism on metabolism and inflammation, there have been few studies to date. In a model using cultured muscle cells, one group tested the hypothesis that PPARδ upregulates FOXO1 activity in muscle, thereby upregulating MAFbx and MuRF1 expression during sepsis and glucocorticoid treatment [52]. Activation of PPARδ in myotubes resulted in increased atrophy along with protein degradation and increased FOXO1 activity. Similar changes induced by dexamethasone (used as an agent to cause atrophy) were prevented by treatment with a PPARδ inhibitor. Furthermore, a PPARδ inhibitor given to dexamethasone-treated or septic rats prevented muscle wasting. These findings appear to support the suggestion that PPARδ may regulate activation of a FOXO1 linked atrophy programme in sepsis-induced muscle wasting. 

Cardiac failure and decreased uptake and oxidation of fatty acids in the heart are common features of severe sepsis [53]. In a mouse model of sepsis [54], LPS administration rapidly caused downregulation of PPARα, PPARδ, as well as isoforms of thyroid hormone receptor (TR) and RXR (which are required for PPAR transcriptional activity) in the heart. There were also concurrent decreases in the expression of key fatty acid transporter/oxidation genes with LPS treatment. Thus, it is possible that these rapid decreases in the expression of key genes, including PPARα and PPARδ, are important in driving the reductions in cardiac fatty oxidation and myocardial dysfunction in sepsis. The potential protective effects of PPARδ agonism have been studied in relation to changes in LPS-induced apoptosis. In cultured rat cardio-myoblast cells, pre-treatment with the PPARδ agonist GW501516 inhibited increased rates of apoptosis induced by LPS, decreased activity of caspase-3 and increased nuclear translocation of NF-kB [55]. Furthermore, GW501516 increased protein expression of haem oxygenase-1 (HO-1), while inhibition of HO-1 reversed the effects of GW501516 on LPS-induced NF-kB activation. Thus, PPARδ also has anti-apoptotic effects during an LPS challenge in cardiac cells, potentially through suppressing NF-kB activation and via HO-1. Whether these observed effects of PPARδ are relevant to skeletal muscle in terms of inflammation and substrate oxidation during sepsis and related conditions remains to be determined. 

To summarise, the effects of PPARδ agonism on skeletal muscle appear to be predominantly related to the switching of fuel utilisation towards increased oxidation of fatty acids, as well as declined carbohydrate oxidation. This can result in impaired function during prolonged muscle contraction where carbohydrate is an obligate fuel. Activation of PPARδ may also induce atrophy-related programmes in skeletal muscle. During sepsis, however, declines in PPAR activity may underlie some of the declines in FFA oxidation in various tissues, indicating that there may be some benefit to PPARδ agonism during these conditions. 

## 4. PPARγ Agonism and Skeletal Muscle Metabolism and Inflammation

As described earlier, PPARγ plays a central role in adipogenesis, and TZDs have been shown to increase insulin-stimulated glucose disposal in T2D patients effectively. The effects of PPARγ agonism on skeletal muscle metabolic regulation remains poorly understood. One study assessed the impact of the PPARγ agonist, Rosiglitazone, on fatty acid transport and oxidation in rat muscle [56]. Seven days of rosiglitazone infusion (1 mg/day) did not alter the rate of fatty acid transport into muscle, but did increase rates of fatty acid oxidation in subsarcolemmal and intermyofibrillar mitochondria. This was accompanied by increases in mitochondrial FAT/CD36 protein, with no changes in citrate synthase or β-HAD activity. The effects of PPARγ activation on lipid metabolism were also studied in human skeletal muscle in vivo [57]. Long-chain fatty acid composition and stearoyl-CoA desaturase 1 (SCD1) were examined following 8 weeks of Rosiglitazone treatment in men with impaired glucose tolerance, with muscle biopsies and hyperinsulinaemic-euglycaemic clamps being carried out before and after rosiglitazone administration. Alongside an increase in insulin sensitivity, SCD1 expression was increased in muscle samples with rosiglitazone treatment. In addition, there was a shift in lipid composition from saturated long-chain fatty acids to unsaturated fatty acids in muscle. These findings clearly indicate a role for PPARγ activation in modulating lipid metabolism in skeletal muscle in vivo. 

The impact of TZDs on skeletal muscle lipid and carbohydrate metabolism has been predominantly studied in relation to animal models of T2D [58,59,60], and patients with T2D [61,62,63]. In one model of obese Zucker rats [59], 6 weeks of rosiglitazone administration improved glucose tolerance in obese rats, while intramuscular triglyceride content, which was higher in obese compared with lean animals, was further increased following rosiglitazone treatment. There were also increases in skeletal muscle diacylglycerol and ceramide with rosiglitazone treatment, indicating that under these conditions, Rosiglitazone increased insulin sensitivity in obese rats, but this was not through reduced fatty acid accumulation in muscle. 

In a study with T2D patients, the effects of three months of rosiglitazone treatment on insulin sensitivity and lipid metabolism were examined during a hyperinsulinaemic-euglycaemic clamp [61]. Insulin-stimulated glucose disposal was improved following rosiglitazone treatment, and reduced plasma fatty acid concentrations and increased extramyocellular lipid levels were observed. Rosiglitazone also promoted increased insulin sensitivity in peripheral adipocytes, indicating that enhanced insulin sensitivity through PPARγ agonism in humans may occur predominantly via improving adipocyte insulin sensitivity, leading to lipid redistribution from insulin-sensitive organs to peripheral adipocytes. In another study with T2D patients, further insight into the mechanisms by which TZDs improved insulin sensitivity in T2D was examined. Pioglitazone treatment for 6 months improved insulin-stimulated glucose disposal in T2D patients. In muscle tissue, there were increases in AMPK and acetyl-CoA carboxylase (ACC) phosphorylation with pioglitazone and increased expression of genes important in fat oxidation and mitochondrial function. These findings suggest some of the mechanisms by which TZDs improve skeletal muscle insulin sensitivity may involve stimulation of AMPK signalling and fat oxidation. 

As described in a previous section, more work is required to determine whether different types of PPAR drug targets may have any potential benefit in alleviating inflammation in sepsis, thereby potentially preventing and/or improving certain deleterious consequences associated with the condition. In relation to specifically PPARγ agonists impacting on targeting inflammation, we previously assessed the impact of Rosiglitazone on muscle carbohydrate and protein metabolism in a rat model of LPS-induced endotoxaemia [64]. Initial work from our laboratory [65,66,67] demonstrated that dysregulation of the Akt/FOXO signalling pathway was important in mediating the development of muscle atrophy during LPS-induced endotoxaemia, specifically through activation of ubiquitin ligases MAFbx and MuRF1. We also proposed that the Akt/FOXO signalling pathway represents a site of molecular crosstalk between insulin and atrophy-related signalling processes during endotoxaemia through FOXO-mediated upregulation of PDK4 and reduced activity of PDC. 

**Figure 2:**
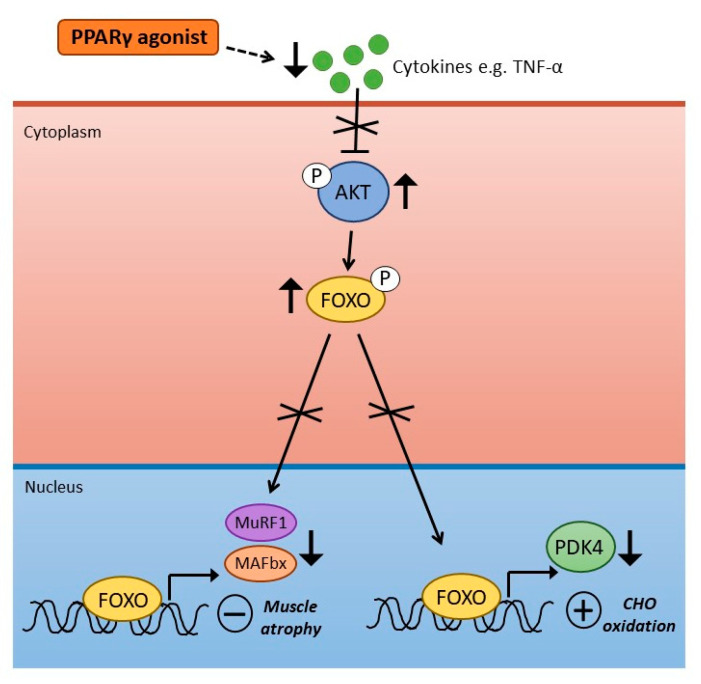
Impact of PPARγ agonism in skeletal muscle during LPS-induced endotoxaemia. Treatment with PPARγ agonists during endotoxaemia suppresses production of pro-inflammatory cytokines (e.g., tumour necrosis factor α: TNF-α). This results in reduced suppression of muscle AKT, and reduced transcriptional activity of Forkhead Box O (FOXO) transcription factors. Reduced activity of FOXO leads to suppression of factors important in increased muscle atrophy (MAFbx and MuRF1) as well as PDK4, a key protein in PDC inhibition.

To assess the effects of PPARγ agonism on muscle protein and carbohydrate metabolism during endotoxaemia, rats were fed standard chow containing Rosiglitazone (8.5 ± 0.1 mg·kg^−1^·day^−1^) for 2 weeks before and during 24 h continuous intravenous infusion of LPS (15 μg·kg^−1^·h^−1^) or saline [64]. In terms of muscle inflammation, Rosiglitazone blunted LPS-induced increases in TNF-α and IL-6 mRNA expression. We also examined the subsequent impact of Rosiglitazone on LPS-induced changes in muscle protein degradation pathways, specifically, ubiquitin-proteasome-mediated protein breakdown. Increased expression of key proteolytic regulators (MAFbx and MurF1 mRNA), and activity of the 20S proteasome, were suppressed in the rosiglitazone-treated group of animals in the presence of endotoxaemia. In carbohydrate oxidation, LPS-induced increases in PDK4 gene expression and muscle lactate content were also suppressed with rosiglitazone administration. Collectively, these findings indicated that there were metabolic benefits of rosiglitazone pre-treatment in this LPS model of endotoxaemia, reflected by blunted muscle cytokine accumulation, muscle protein loss and lactate accumulation (Figure 2).

While few studies have assessed the impact of PPARγ agonists on skeletal muscle inflammation and metabolism during inflammatory disorders such as sepsis, there has been some work surrounding the protective effects of PPARγ on myocardial dysfunction in sepsis. One study in mice (using LPS administration as a sepsis model) investigated whether reduced fatty acid oxidation is the underlying cause for cardiac dysfunction in sepsis [68]. LPS administered to mice rapidly decreased cardiac fatty acid oxidation in conjunction with inducing cardiac dysfunction, while gene expression of PPARγ was downregulated. Moreover, activation of PPARγ using a transgenic mouse model (cardiomyocyte-specific PPARγ expression induced by the alpha-myosin heavy chain promoter) protected against cardiac dysfunction induced by LPS, while fatty acid oxidation was not reduced with LPS exposure in these animals. Interestingly, the expression of inflammation-related genes (IL-1α, IL-1β, IL-6 and TNF-α) in response to LPS treatment was similar to wild-type mice. Rosiglitazone administration in wild-type mice similarly increased fatty acid oxidation, improved cardiac function after treatment with LPS and improved survival, despite not suppressing the expression of cardiac markers of inflammation. These findings are, therefore, promising in terms of the use of PPARγ agonists in sepsis treatment. Still, more work should be done on their mechanism of action and delineating whether their beneficial effects occur through their anti-inflammatory actions.

In relation to myocardial dysfunction, a separate study in rats assessed the mechanism of PPARγ-mediated cardiac protective effects in sepsis [69]. Using rats subjected to caecal ligation and puncture (CLP), a PPARγ agonist (Rosiglitazone) and antagonist (T0070907) were used. The model of sepsis used resulted in significant impairments in cardiac function, with evidence of tissue apoptosis, necrosis and upregulated proinflammatory cytokines. Activation of PPARγ prevented these changes while blocking its activity exacerbated the differences and further reduced survival rates. These results provide other evidence that Rosiglitazone can exert beneficial effects related to reduced cardiac inflammation and cell death. A separate recent study further examined the effects of Rosiglitazone on sepsis-induced myocardial dysfunction in relation to the NF-κB pathway [70]. Here, a model of sepsis was established using female Sprague-Dawley rats, after which one group was administered 3 mg/mg rosiglitazone (daily, for 3 days). Rosiglitazone successfully decreased the number of apoptotic cells in septic animals, while in myocardial tissues, Rosiglitazone lowered TNF-α expression and activity of NF-κB. 

To summarise, agonism of PPARγ in vivo appears to improve insulin sensitivity and may also increase fatty acid oxidation in skeletal muscle. However, improved insulin sensitivity may not be related to reductions in fatty acid accumulation in muscle tissue under certain conditions. In relation to inflammatory conditions such as sepsis, PPARγ agonists effectively suppress pro-inflammatory cytokine production and appear to be beneficial in alleviating organ injury and dysfunction, which has promising potential for therapeutic development. 

## 5. Conclusions and Future Perspectives

To conclude, the PPAR family of transcription factors have wide-ranging critical regulatory roles in skeletal muscle and other tissues, from inflammation to fuel selection and contractile function. In terms of PPARδ, evidence suggests that the agonism of PPARδ appears to be primarily related to the switching of substrate utilisation towards increasing the use of fatty acids. In contrast, PPARδ agonism can impair muscle contractile function by inhibiting carbohydrate oxidation during muscle contraction, where carbohydrate is an obligate fuel. Conversely, there may be benefits to PPARδ agonism during certain inflammatory conditions, such as sepsis, since declines in PPAR activity may underlie some of the reductions in FFA oxidation. Similarly, agonism of PPARγ in vivo appears to be an effective anti-inflammatory strategy during sepsis and has proven beneficial in improving organ/tissue function in pre-clinical models. Blunting muscle cytokine accumulation during endotoxaemia in rodents has also been demonstrated to result in metabolic benefits via reduced muscle wasting and lactate accumulation. 

Moving forwards, more studies will be required to better define the mechanistic roles of all the PPARs in different physiological and pathophysiological conditions. The potential benefits of PPAR agonism on skeletal muscle protein and carbohydrate/lipid metabolism during sepsis and other inflammatory conditions remains under-investigated and is, therefore, a promising area of research opportunity. Studies using dual or pan-PPAR agonists could also widen the therapeutic potential of these compounds, while cross-tissue studies will be important in evaluating potential off-target effects. Nevertheless, with ongoing drug developments and a greater understanding of the wide-ranging functions of PPARs, these transcription factors will undoubtedly remain critical therapeutic targets for a multitude of metabolic and inflammatory conditions.

## Figures and Tables

**Table 1:** Regulation of lipid and carbohydrate metabolism by PPARs in skeletal muscle, adipose tissue and liver.

	Skeletal Muscle	Liver	Adipose
PPARδ	+ FA oxidation −carbohydrate oxidation	+ FA oxidation − lipogenesis	+ FA oxidation
PPARγ	+ FA oxidation + glucose uptake	+ lipogenesis + lipid storage − glucose production	+ adipogenesis + lipogenesis + lipid storage + adipokine production
PPARα	+ FA oxidation	+ FA oxidation − lipid storage	

## Data Availability

Not applicable.
